# S90. IMPLICIT PROCESSING OF BODILY EMOTIONS IN SCHIZOPHRENIA

**DOI:** 10.1093/schbul/sby018.877

**Published:** 2018-04-01

**Authors:** Michal Hajdúk, Hans Klein, Emily Bass, Amy Pinkham

**Affiliations:** 1 Comenius University; 2 University of Texas at Dallas; 3 University of Texas at Dallas,University of Texas Southwestern Medical School

## Abstract

**Background:**

Explicit emotion recognition from faces is severely impaired in patients with schizophrenia; however, implicit processing of facial emotion appears to be intact and comparable to healthy control individuals (Shasteen et al., 2016). Social cues are not restricted to facial expressions, and body posture makes substantial contributions to nonverbal communication (de Gelder, 2006). The role of body perception in social processing among individuals with schizophrenia has not yet been studied. The aim of the present study was to evaluate whether intact implicit processing of emotion in patients with schizophrenia spectrum disorders extends to body cues devoid of facial information.

**Methods:**

Fifty-three patients diagnosed with schizophrenia spectrum disorders and 34 matched healthy controls completed the Affect Misattribution Task, a paradigm in which affective responses to primes are projected onto neutral targets. Primes consisted of pictures from The Bodily Expressive Action Stimulus Test and included 24 images each of bodies expressing happy, angry, and neutral expressions. Participants were asked to indicate whether neutral targets (i.e., Chinese symbols) were more or less threatening than average symbol. Scores on the Paranoia Scale and PANSS Suspiciousness item were used for measuring levels of paranoid ideation.

**Results:**

A repeated measures ANOVA with prime type as the within-subjects factor and group membership as the between-subjects factor revealed significant main effects for prime type (F(2,170)= 16.722, p < .001, η2 = .164) and group (F(1,85)= 5.704, p = .019, η2 = .063) such that patients identified more targets as threatening and, across both groups more targets were identified as threatening in the anger condition relative to the neutral and positive condition. The interaction was not significant (F(2,170) =.142, p = .868, η2 =.002). In patients, the total number of threatening responses was positively correlated with self-reported paranoid ideation measured by the Paranoia Scale (r = .391, p = .004). However, the association between PANSS Suspiciousness ratings and number of threatening responses was not significant (r = .168, p = .229).

**Discussion:**

In both groups, angry bodies were rated as more threatening than neutral and happy expressions, which suggest that patients have intact implicit processing of emotions from body cues. This parallels previous findings of intact implicit processing in facial emotion perception. Patients also tended to rate stimuli as more threatening on average, which may be partially explained by higher levels of paranoid ideation in this group. Results will be discussed in relationships to threat processing theories.

Research was supported by Slovak Research and Development Agency no: APVV-15–0686 and internal funding provided by The University of Texas at Dallas.

